# Long-Acting Thioredoxin Ameliorates Doxorubicin-Induced Cardiomyopathy via Its Anti-Oxidative and Anti-Inflammatory Action

**DOI:** 10.3390/pharmaceutics14030562

**Published:** 2022-03-03

**Authors:** Ryota Murata, Hiroshi Watanabe, Hiroto Nosaki, Kento Nishida, Hitoshi Maeda, Motohiro Nishida, Toru Maruyama

**Affiliations:** 1 Department of Biopharmaceutics, Graduate School of Pharmaceutical Sciences, Kumamoto University, Kumamoto 862-0973, Japan; 217y2002@st.kumamoto-u.ac.jp (R.M.); 184p1013@st.kumamoto-u.ac.jp (H.N.); spbv8d99@gmail.com (K.N.); maeda-h@kumamoto-u.ac.jp (H.M.); tomaru@gpo.kumamoto-u.ac.jp (T.M.); 2 Department of Physiology, Graduate School of Pharmacological Sciences, Kyushu University, Fukuoka 812-8582, Japan; nishida@phar.kyushu-u.ac.jp; 3 Division of Cardiocirculatory Signaling, National Institute for Physiological Sciences and Exploratory Research Center on Life and Living Systems, National Institutes of Natural Sciences, Okazaki 444-8787, Japan

**Keywords:** albumin fusion, thioredoxin, oxidative stress, inflammation, cardiomyopathy, doxorubicin

## Abstract

Although the number of patients with heart failure is increasing, a sufficient treatment agent has not been established. Oxidative stress and inflammation play important roles in the development of myocardial remodeling. When thioredoxin (Trx), an endogenous anti-oxidative and inflammatory modulator with a molecular weight of 12 kDa, is exogenously administered, it disappears rapidly from the blood circulation. In this study, we prepared a long-acting Trx, by fusing human Trx (HSA-Trx) with human serum albumin (HSA) and evaluated its efficacy in treating drug-induced heart failure. Drug-induced cardiomyopathy was created by intraperitoneally administering doxorubicin (Dox) to mice three times per week. A decrease in heart weight, increased myocardial fibrosis and markers for myocardial damage that were observed in the Dox group were suppressed by HSA-Trx administration. HSA-Trx also suppressed the expression of atrogin-1 and myostatin, myocardial atrophy factors in addition to suppressing oxidative stress and inflammation. In the Dox group, a decreased expression of endogenous Trx in cardiac tissue and an increased expression of macrophage migration inhibitory factor were observed, but these changes were restored to normal levels by HSA-Trx administration. These findings suggest that HSA-Trx improves the pathological condition associated with Dox-induced cardiomyopathy by its anti-oxidative/anti-inflammatory and myocardial atrophy inhibitory action.

## 1. Introduction

Heart failure is caused by drug-induced cardiomyopathy, ischemic heart disease or hypertrophic cardiomyopathy. In either case, the pathological condition progresses through morphological changes (myocardial remodeling) in the myocardium caused by the damage such as oxidative stress and inflammation [1]. Myocardial atrophic remodeling that has attracted attention in recent years is induced through the induction of myocardial atrophy factors such as the muscle-specific ubiquitin ligase atrogin-1 and myostatin [2]. The fibrosis that occurs during myocardial remodeling also increases the risk of death in patients with heart failure [3]. Although myocardial remodeling is an important therapeutic target for the treatment of heart failure pathology, an effective therapeutic agent for treating myocardial remodeling has not yet been developed.

Oxidative stress and inflammatory response play a central role in the progression of myocardial remodeling. During the onset and progression of this pathology, an increase in the activity of NADPH oxidase (Nox) or xanthine oxidase, eNOS uncoupling and mitochondrial damage induces the production of reactive oxygen species (ROS). At the same time, the weakening of anti-oxidative defense systems such as thioredoxin (Trx), superoxide dismutase (SOD) and glutathione (GSH) results in a substantial increase in oxidative stress. In fact, it has been reported that Nox is activated and the expressions of Trx, SOD and GSH are decreased in heart failure mice [4,5,6,7,8] such as doxorubicin (Dox)-induced cardiomyopathy [9] and diabetic heart failure [4]. Regarding the inflammatory reaction, the infiltration of inflammatory cells into the heart is induced, and the production of inflammatory cytokines is increased accordingly. In addition, the macrophage migration inhibitory factor (MIF) contributes to the inflammatory response. MIF is produced from stressed cardiomyocytes and macrophages, and acts on macrophages in a paracrine and autocrine manner to induce the expression of inflammatory cytokines. In fact, it was reported that MIF knock-out mice [10] or the administration of an anti-MIF antibody to mice [11] showed a suppressing effect on ischemic heart disease. Based on a comprehensive interpretation of these findings, we hypothesized that a molecule that has anti-oxidative and anti-inflammatory effects and also has the ability to control MIF would be a promising novel therapeutic agent for the treatment of heart failure.

Thioredoxin (Trx) is a redox-regulating protein that is produced ubiquitously in the body and functions as an anti-oxidative/inflammatory modulator [12,13]. In addition to its inherent direct superoxide and hydroxyl radical scavenging activity [14], Trx itself is translocated to the extracellular space when oxidative stress is stimulated [15], where it suppresses neutrophil migration/infiltration and extravasation at inflammatory sites [16,17,18,19] and also suppresses the expression and secretion of MIF [17,20]. In addition, Trx acts directly on infiltrative macrophages, thus promoting the production of anti-inflammatory cytokines by inducing M2 conversion of macrophages [21]. It has been reported that mice that overexpress Trx show a suppressing effect on heart failure models such as Dox-induced cardiomyopathy [22], ischemic heart disease [23] and angiotensin II-induced cardiac hypertrophy [24]. However, since Trx has a molecular weight of 12 kDa, it is readily removed from the circulation by glomerular filtration. Its short blood retention and low utilization to target organs are bottleneck for its clinical application. In order to overcome this problem, we report on the production of Trx fused with human serum albumin (HSA) (HSA-Trx) using a Pichia expression system [25,26,27,28,29,30,31,32,33]. The results of pharmacokinetic experiments in mice confirmed that the elimination half-life of HSA-Trx from blood was extended by 10 times or more as compared to that of Trx [25]. HSA-Trx has been demonstrated to exert a therapeutic effect on oxidative stress-induced organ damage in the kidney, lung and liver [25,26,27,28,29,30,31,32,33].

In this study, we investigated the cardioprotective effect of HSA-Trx using Dox-induced cardiomyopathy mice, which are animal models of drug-induced heart failure. The use of smaller repeated doses in animals adequately mimics chronic myocardial alterations in patients [34]. The findings of our study indicate that the administration of HSA-Trx alleviates Dox-induced myocardial damage by its anti-oxidative/anti-inflammatory and myocardial atrophy inhibitory action.

## 2. Materials and Methods

### 2.1. Materials

Yeast nitrogen base with ammonium sulfate without amino acids was purchased from Difco Laboratories, Inc. (Detroit, MI). Hipolypepton was purchased from Nippon Shinyaku (Kyoto, Japan). Aquacide II was purchased from Merck Millipore (Burlington, MA, USA). Blue Sepharose 6 Fast Flow column and HiTrap Phenyl HP column were purchased from GE Healthcare Japan (Tokyo, Japan). Sal 1 and RNAiso Plus were purchased from Takara Bio. (Shiga, Japan). A 10% formalin neutral buffer solution was purchased from FUJIFILM Wako Pure Chemical (Osaka, Japan). Heparin sodium was purchased from Mochida Pharmaceutical (Tokyo, Japan). 4′,6-diamidino-2-phenylindole (DAPI) solution was purchased from Invitrogen (Waltham, MA, USA). Masson’s trichrome and Marinol were purchased from Muto Pure Chemical (Fukuoka, Japan). All reagents and solvents were commercially available, special-grade products, and water as a solvent was ion-exchanged water or Milli-Q water.

### 2.2. Preparation and Purification of HSA-Trx

The Pichia Expression Kit was purchased from Invitrogen (Carlsbad, CA, USA). The production and purification of HSA-Trx were performed as described in a previously reported method [35].

### 2.3. Mice Model of Dox-Induced Heart Failure

The 7-week-old male C57BL/6J WT mice (SLC, Japan) were randomized by body weight. The heart failure mice were induced by the intraperitoneal administration of Dox (3 mg/kg) three times per week for 2~4 weeks [36]. At 2 weeks after Dox administration, HSA-Trx (400 nmol/kg) was intravenously administered three times per week for 2 weeks (HSA-Trx group). The control group was injected with an equivalent amount of PBS (10 mL/kg) (Dox-PBS group). The mice were sacrificed at 4 weeks after Dox administration. Body weight of each mouse was monitored during the experiments. All animal experiments involved procedures that had been previously approved by the experimental animal ethics committee at Kumamoto University and all methods were performed in accordance with the relevant guidelines and regulations.

### 2.4. Measurement of Myocardial Damage

Blood was collected from mice at days 14 and 28 after Dox administration. Creatine phosphokinase (CPK) and lactate dehydrogenase (LDH) as markers of myocardial damage were measured using FUJI DRI-CHEM (Fujifilm, Japan).

### 2.5. Histological Analysis

The accumulation of collagen (fibrosis) in the heart was detected by picrosirius red staining. Staining was performed by following the previously described protocol [37]. Immunofluorescence staining of 8-OHdG (Rabbit anti-8-OHdG polyclonal antibody; BIOSS, cat#: bs-1278R, Woburn, MA, USA), as oxidative stress marker, was performed as described in a previous report [32]. Heart sections with thicknesses of 4 µm were prepared and then observed using a BZ-X710 microscope (Keyence, Japan) (original magnification power × 200). The images were randomly acquired, with 10 to 14 high-power fields collected for each mouse and then quantified within them.

### 2.6. qRT-PCR

Isolation of total RNA from heart tissue and the quantitative RT-PCR were performed as previously described [37]. Each primer sequence is shown below (Table S1, Supplementary Material).

### 2.7. Western Blotting

Western blotting was performed following a previous report [35]. Heart tissue was homogenized with RIPA buffer (150 nM NaCl, a 1% Nonidet P-40, 1% protease inhibitor, 10 mM Tris/HCl (pH7.4)) and separated by 12.5% SDS-PAGE. Proteins were transferred to a PVDF membrane and then incubated with the primary antibody (Rabbit anti-thioredoxin polyclonal antibody; CST Japan, cat#: 2429, Tokyo, Japan, Rabbit anti-MIF polyclonal antibody; GeneTex, cat#: GTX101162, Irvine, CA, US) at 4 °C overnight. The secondary antibody (Mouse anti-rabbit IgG-HRP; Santa Cruz Biotechnology, cat#: sc-2357, Dallas, TX, USA) was incubated at room temperature for 1 hr. Each band was detected by LAS 4000mini (GE Healthcare, UK Ltd., Buckinghamshire, England) and quantified using the ImageJ software (National Institutes of Health).

### 2.8. Isolation and Cell Culture of Neonatal Rat Cardiomyocytes (NRCMs)

The heart was excised from a 2-day-old SD rat and exsanguinated with PBS. This experiment was essentially performed as described in a previous report [38]. The heart was cut in half and allowed to shake overnight at 4 °C in a flask at 40 rpm with 0.25% trypsin EDTA. The supernatant was incubated with DMEM (10% hyclone fetal bovine serum, characterized (FBS) (GE Healthcare, cat#: SH30071.03, Chicago, IL, USA) that included a penicillin–streptomycin mixed solution (Nacalai Tesque, cat#: 26253-84, Kyoto, Japan) in D-MEM High glucose) at 37 °C for 10 min. The supernatant was then filtered through a cell strainer and cultured at 37 °C with shaking. The sample was centrifuged, and the supernatant was seeded on a plate. After removing the fibroblasts, the supernatant was seeded in a plate and the cells were treated as NRCMs. NRCMs were cultured in DMEM containing 10% FBS, penicillin (100 U/mL) and streptomycin (100 mg/mL) under 37 °C and an atmosphere of 5% CO2. The differentiation of NRCMs was induced by culturing in DMEM containing taurine, penicillin (100 U/mL) and streptomycin (100 U/mL) for 2 days.

### 2.9. Evaluation of Cell Size in NRCMs

Cell size analysis was assessed by fluorescence staining with phalloidin-Alexa 488 (Thermo Fisher Scientific, cat#: A12379, Waltham, MA, USA). NRCMs were seeded onto 24 well plates and cultured overnight. After a 2 day period of differentiation, Dox (3 mmol/L) and HSA-Trx (1–10 mmol/L) in D-MEM were added and incubated at 37 °C for 12 h. After washing with PBS, 4% PFA in PBS was added and incubated for 10 min at room temperature. Permeabilization was achieved by adding 0.5% TrironX-100 in a Tris-buffered saline solution (TBS). After washing with 0.1% TritonX-100 in TBS and blocking with 0.1% TrironX-100, 1% BSA in TBS, the antibody reaction was performed by phalloidin-Alexa 488 in blocking solution. The cells were observed by a fluorescence microscope (BZ-X710; Keyence, Osaka, Japan) and analyzed using the Image J software (National Institutes of Health and LOCI, University of Wisconsin, Madison, WI, USA).

### 2.10. Measurement of Intracellular ROS Production

Intracellular ROS levels were measured using the CM-H_2_DCFDA. NRCMs were seeded on 35 mm glass bottom plates and cultured overnight. After differentiation for 2 days, Dox (3 mmol/L) and HSA-Trx (1–10 mmol/L) were added to the medium, and the cells were cultured for 12 h. After washing with HBSS, the cells were cultured in 1 mmol/L CM-H_2_DCFDA in HBSS for 30 min, then washed and observed with a fluorescence microscope. Image scoring was performed using the ImageJ software (National Institutes of Health and LOCI, University of Wisconsin, Madison, WI, USA).

### 2.11. Statistical Analysis

Data from animal and cell studies were compared by analysis of variance followed by Tukey’s multiple comparison. All results are expressed as the mean ± SE of the indicated experiments. A P value < 0.05 was statistically significant.

## 3. Results

### 3.1. Effect of HSA-Trx on Myocardial Damage and Fibrosis in the Dox-Induced Cardiomyopathy Model

**Figure 1:**
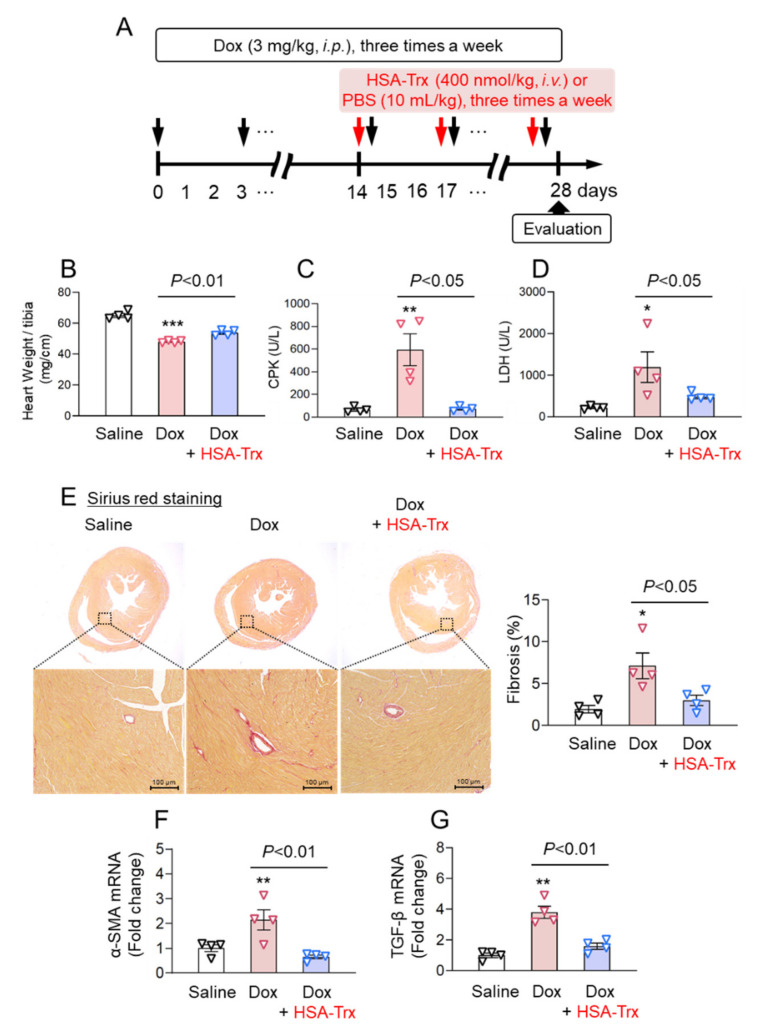
Effect of HSA-Trx on myocardial atrophy and fibrosis in the heart of Dox-induced cardiomyopathy model mice. (**A**) Experimental protocol used for the evaluation of HSA-Trx on Dox-induced cardiomyopathy model mice: Dox-induced heart failure was induced by an intraperitoneal injection of 3 mg/kg Dox three times per week for 4 weeks. HSA-Trx (400 nmol/kg) was administered i.v. three times a week at 2 weeks after the first injection of Dox. An equivalent amount of PBS (10 mL/kg) was administered to the saline injection group (normal mice) and the Dox injection group. (**B**) Heart weight at 4 weeks after the first injection of Dox. Change in the level of (**C**) creatine phosphokinase (CPK) and (**D**) lactate dehydrogenase (LDH) at 4 weeks after the first injection of Dox. (**E**) Representative photomicrographs of Sirius red-stained heart sections at 4 weeks after the first injection of Dox. Original magnification: ×200. Scale bars represent 100 mm. Image analysis was performed to quantify the area of Sirius red staining. (**F**) α-SMA and (**G**) TGF-β mRNA expression in the heart at 4 weeks after the first injection of Dox were determined by real-time qPCR. Results are the means ± S.E. (*n* = 4). * *p* < 0.05, ** *p* < 0.01, *** *p* < 0.001 compared with the saline injection group (normal mice).

Dox-induced cardiomyopathy model mice were developed by the intraperitoneal injection of Dox (3 mg/kg) three times per week for 4 weeks [36] (Figure S1). We evaluated the therapeutic effect of HSA-Trx on Dox-induced cardiomyopathy model mice (Figure S1). A decrease in heart weight (Figure S1B), an increase in CPK (Figure S1C) and cardiac fibrosis (Figure S1D) were observed at 2 weeks after the start of Dox administration and these changes were further increased at 4 weeks after Dox administration. At 2 weeks after Dox administration, HSA-Trx was administered three times per week for another two weeks (HSA-Trx group). As a control group, phosphate-buffered saline (PBS) was administered in the same volume as HSA-Trx (Dox group). Although weight loss was observed in the Dox-PBS group, weight loss was observed to be suppressed in the HSA-Trx group from day 26 (Figure S2). In addition, HSA-Trx administration significantly suppressed the decreased heart weight as observed in the Dox group (Figure 1B). Serum CPK and LDH levels, as markers of myocardial dysfunction, were significantly increased in the Dox group, while HSA-Trx administration caused a significant suppression in these levels (Figure 1C,D). These results suggest that HSA-Trx ameliorates Dox-induced myocardial damage.

Myocardial fibrosis was evaluated histologically by Sirius red staining. Fibrosis formation was observed in the Dox group. However, the administration of HSA-Trx resulted in a significant improvement in the fibrotic area (Figure 1E). Figure 1E also showed the representative overall picture of heart section. The data indicated that the decreased heart size induced by Dox was recovered by the administration of HSA-Trx. In addition, the expression of a-SMA and TGF-b, markers of fibroblast activation, was increased in the Dox group, but both a-SMA and TGF-b were significantly suppressed by HSA-Trx administration (Figure 1F,G). These findings indicate that HSA-Trx prevents myocardial fibrosis during the process of myocardial remodeling.

### 3.2. Anti-Oxidative and Anti-Inflammatory Effect of HSA-Trx on the Dox-Induced Cardiomyopathy Model

**Figure 2:**
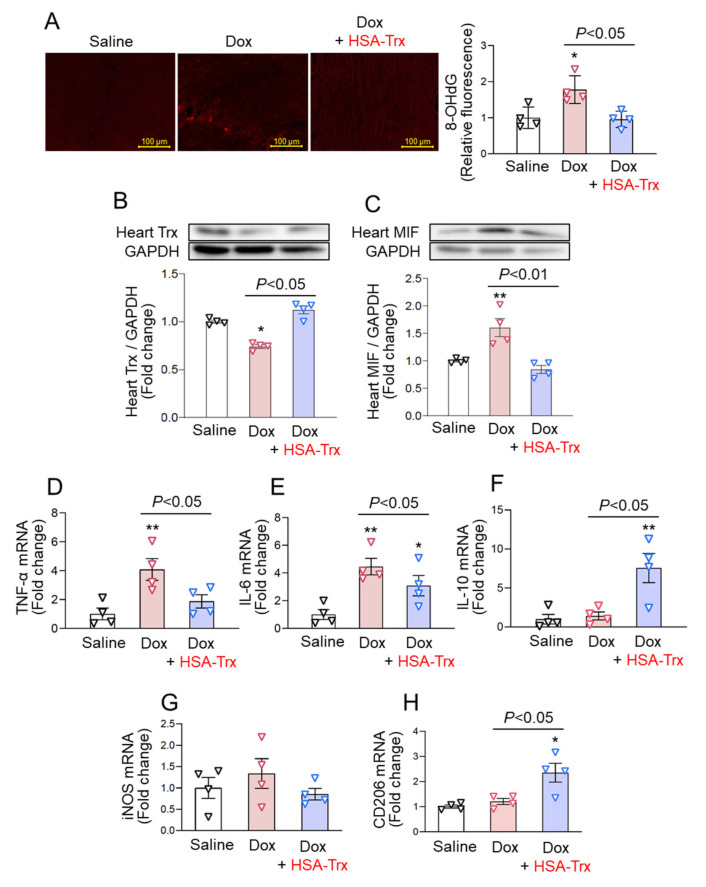
The effect of HSA-Trx on myocardial oxidative stress and inflammation in the hearts of Dox-induced cardiomyopathy model mice. (**A**) Representative photomicrographs of immunostaining for myocardial 8-OHdG (8-hydroxy-2′-deoxygenase) are shown at 4 weeks after the first injection of Dox. Original magnification: ×400. Scale bars represent 100 mm. Image analysis was performed to quantify the extent and intensity of 8-OhdG staining. (**B**) Trx and (**C**) MIF expression in the heart at 4 weeks after the first injection of Dox were assessed by Western blotting. (**D**) TNF-α, (**E**) I IL-6, (**F**) IL-10, (**G**) iNOS and (**H**) CD206 mRNA expression in the heart at 4 weeks after the first injection of Dox were determined by real-time qPCR. PBS or HSA-Trx (400 nmol/kg) was administered i.v. three times a week from 2 weeks after the first injection of Dox. Results are the means ± S.E. (*n* = 4). * *p* < 0.05, ** *p* < 0.01 compared with the saline injection group (normal mice).

Oxidative stress and inflammation play a central role in the progression of myocardial remodeling. We evaluated the production of 8-OHdG, a marker of oxidative stress, by immunofluorescence staining in myocardial tissue. The levels of 8-OHdG in myocardial tissue were significantly increased in the Dox group, whereas HSA-Trx administration significantly suppressed this increase (Figure 2A). Interestingly, consistent with the previous reports that cardiac Trx expression was downregulated in the heart failure state [4,5], the expression of endogenous Trx expression in cardiac tissue was decreased in the Dox group. In contrast, the decrease in cardiac Trx expression was recovered in the HSA-Trx group (Figure 2B and Figure S3). Previous reports have shown that the anti-inflammatory effects of Trx involve the inhibition of MIF [17,20,39] and the M2 polarization of macrophages [40]. In this study, MIF expression in myocardial tissue was increased in the Dox group, but this increased expression was suppressed to the normal levels by the administration of HSA-Trx (Figure 2C and Figure S3). The increased expression of pro-inflammatory cytokines such as IL-6 and TNF-a in the Dox group was significantly suppressed (Figure 2D,E), and IL-10, an anti-inflammatory cytokine, was significantly upregulated by the administration of HSA-Trx (Figure 2F). Supporting these results, there was no change in iNOS, an M1 macrophage marker, while the level of CD206, an M2 macrophage marker, was significantly increased in the HSA-Trx group (Figure 2G,H). These results suggest that the anti-inflammatory effect of HSA-Trx involves the inhibition of MIF expression and the promotion of the M2 macrophages. These collective results suggest that HSA-Trx exerted anti-oxidative and anti-inflammatory effects on the Dox-induced cardiomyopathy model, then it inhibited myocardial remodeling, which is important for the development of the pathology of heart failure.

### 3.3. Anti-Myocardial Atrophy Effect of HSA-Trx on the Dox-Induced Cardiomyopathy Model

**Figure 3:**
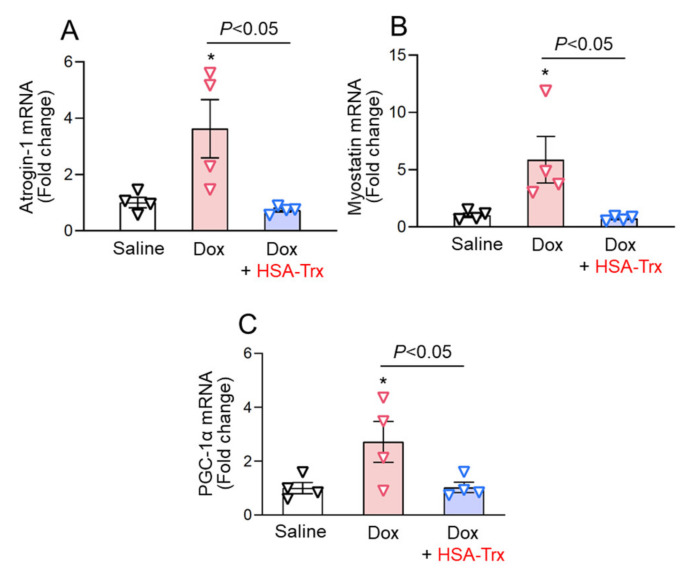
Effect of HSA-Trx on myocardial atrophy-related genes in the heart of Dox-induced cardiomyopathy model mice. (**A**) Atrogin-1, (**B**) myostatin and (**C**) PGC-1a mRNA expression in the heart at 4 weeks after the first injection of Dox were determined by real-time qPCR. PBS or HSA-Trx (400 nmol/kg) was administered i.v. three times a week from 2 weeks after the first injection of Dox. Results are the means ± S.E. (*n* = 4). * *p* < 0.05 compared with the saline injection group (normal mice).

In Dox-induced cardiomyopathy, an increased expression of atrogin-1, a muscle atrophy factor, contributes to the development of cardiomyopathy [2,9]. It was also reported that the myocardial-specific overexpression of myostatin, which is also a muscle atrophy factor, induced myocardial atrophy, fibrosis and an impaired cardiac function [41,42]. In this study, we also observed an increase in the mRNA expression of atrogin-1 and myostatin in myocardial tissue in the Dox group, whereas these changes were suppressed by HSA-Trx administration to the same levels as for the normal (saline) group (Figure 3A,B). Since PGC-1a contributes to the maintenance of mitochondrial biosynthesis and function, it is an important factor for the energy metabolism in the heart. Consistent with previous reports [43], the expression of PGC-1a was upregulated with myocardial damage in the Dox group, but was significantly suppressed in the HSA-Trx group (Figure 3C).

### 3.4. Effect of HSA-Trx on Dox-Induced Cardiomyocyte Atrophy and ROS Production

**Figure 4:**
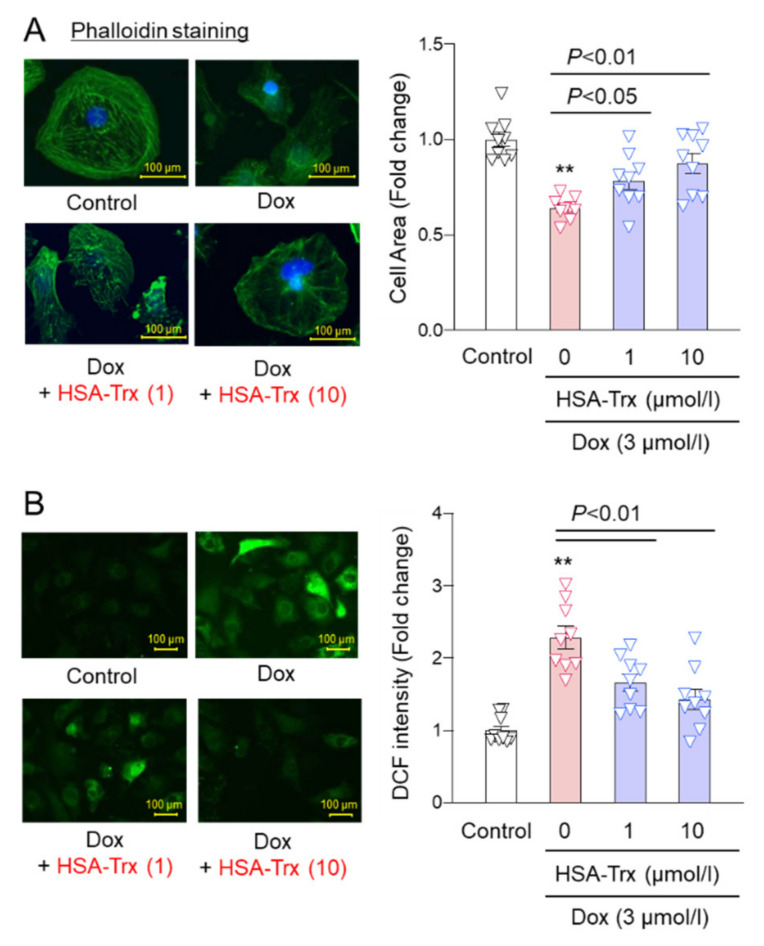
Effect of HSA-Trx on cell shrinkage and cellular ROS level in Dox-treated NRCMs: (**A**) Dox-induced cell shrinkage was determined by phalloidin staining in NRCMs. The phalloidin fluorescence was excited by illumination at 488 nm and then observed at room temperature using a confocal laser microscopy. NRCMs were also treated with DAPI (blue). Original magnification: ×25. Scale bar represents 100 mm. (**B**) Production of ROS in NRCMs was measured using CM-H_2_DCFDA, an ROS-sensitive fluorescent dye. Image analysis was performed to quantify the extent and intensity of DCF staining. Original magnification: ×10. Scale bar represents 100 µm. Results are the means ± S.E. (*n* = 9). ** *p* < 0.01 compared with control.

In an in vivo mice model, HSA-Trx was shown to exert a preventive effect on myocardial atrophy via its anti-oxidative and anti-inflammatory effects. Therefore, we investigated the effect of HSA-Trx on Dox-induced cardiomyocyte atrophy in vitro. Neonatal rat cardiomyocytes (NRCMs) isolated from 2-day-old SD rats were co-incubated with 3 mM of Dox and 1–10 mM of HSA-Trx, then phalloidin staining was performed. As a result, cardiomyocyte atrophy was observed at 12 h after the addition of Dox and the atrophy was significantly inhibited by the co-addition of HSA-Trx (Figure 4A). In parallel, Dox-induced ROS production in cardiomyocytes was also prevented by the presence of HSA-Trx (Figure 4B).

## 4. Discussion

It is important to suppress myocardial remodeling and interstitial fibrosis in the treatment of heart failure, and oxidative stress and inflammatory reactions are involved in this pathological development process [9]. In this study, we evaluated the efficacy of HSA-Trx, a long-acting anti-oxidative/inflammatory modulator, in Dox-induced heart failure mice. Our findings indicate that the administration of HSA-Trx improves Dox-induced myocardial atrophy and interstitial fibrosis. Concerning the mechanism of its action, HSA-Trx showed anti-oxidative and anti-inflammatory effects. It therefore appears that HSA-Trx could be useful as a therapeutic agent for the treatment of cardiomyopathy.

Although it has been reported that the onset of Dox-induced cardiomyopathy is caused by oxidative stress produced by Dox itself [44], Shimauchi et al. recently reported that Dox induces the expression of the Ca^2+^ transient receptor potential canonical (TRPC3), which is present in the myocardial cell membrane, and enhances ROS production from Nox2, resulting in the atrophy of cardiomyocytes [9]. In this study, the administration of HSA-Trx suppressed oxidative stress in myocardial tissue (Figure 2A) and ROS production in cardiomyocytes (Figure 4B). Trx has ROS scavenging ability through a coordinated reaction with its own free thiol group and peroxiredoxin (Prx), and also exerts an anti-oxidative effect by reducing redox-related proteins [45]. Since HSA-Trx retains the anti-oxidative capacity of Trx [25], this indicates that the ROS-scavenging action of Trx contributes to the effect on Dox-induced cardiomyopathy.

Interestingly, during heart failure, the endogenous Trx expression in myocardial tissue is decreased [4,5]. Consistent with this observation, a decrease in Trx expression in myocardial tissue was also observed in these Dox-induced cardiomyopathy mice (Figure 2B). On the other hand, in Trx-overexpressing mice, the progression of various types of heart failure (Dox-induced cardiomyopathy [22], ischemic heart disease [23] and angiotensin II-induced cardiac hypertrophy [24]) was suppressed. These findings highlight the importance of endogenous Trx expression in the pathology of heart failure. In normal conditions, Trx functions intracellularly, but during oxidative stress, it is also secreted extracellularly and its intracellular levels are then decreased. In fact, Kasuno et al. reported that treating human proximal tubular epithelial cell lines with hydrogen peroxide resulted in an increased secretion of Trx into culture supernatants, and that the secretion was suppressed by the addition of anti-oxidants [15]. Such oxidative stress-responsive extracellular translocation appears to be unique for Trx and is not found in SOD and catalase. The physiological significance of this phenomenon currently remains unclear, but suppressing extracellular oxidative stress and the inflammatory reaction may be important issues. This leads to the speculation that the decrease in Trx expression in myocardial tissue observed in this study (Figure 2B) may be due to the migration of Trx to the outside of cardiomyocytes due to the persistent oxidative stress and inflammation induced by Dox. This study showed that HSA-Trx administration was found to restore the endogenous Trx levels to normal levels in myocardial tissue (Figure 2B). This may be due to the suppression in extracellular oxidative stress and the inflammatory reaction by the extracellular HSA-Trx, so that the intracellular Trx was not secreted to the outside of the cell and was retained inside the cell. In this study, the changes of the expression levels of other Trxs, such as Trx2 or the other redox regulating genes such as Sod, Cat, Txnip and Akap [23], may also be involved in the effect of HSA-Trx. This experimental evidence would be useful in future investigations.

In the process of myocardial remodeling, MIF is generally thought to be an important factor in the progression of pathological conditions because its expression is increased and the inflammatory reaction is promoted in heart failure such as ischemic heart disease [10]. In this study, the administration of HSA-Trx suppressed the increase in MIF expression in myocardial tissue associated with Dox administration (Figure 2C). MIF is produced by stressed cardiomyocytes and macrophages, and it acts on macrophages to induce the expression of inflammatory cytokines [46]. In this study, an increased expression of inflammatory cytokines (IL-6 and TNF-a) was observed in the myocardial tissue of Dox-treated mice (Figure 2D,E), and the administration of HSA-Trx significantly suppressed the expression of these inflammatory cytokines (Figure 2D,E). On the other hand, it has been reported that the cardiovascular protective effect of Trx on arteriosclerotic mice is related to M2 conversion of macrophages (phenotypic change to anti-inflammatory macrophages) [21]. In this study, we also found that the expression of IL-10, which is an anti-inflammatory cytokine, and CD206, which is known to be an M2-like macrophage marker, was increased in the HSA-Trx-administered group (Figure 2F,H). These data suggest that the suppression of MIF expression and M2 conversion of macrophage could be involved in the preventive effect of HSA-Trx on myocardial remodeling.

In recent years, apoptosis signal-regulating kinase 1 (ASK1) has attracted attention as a regulator of myocardial remodeling [47]. ASK1 regulates various vital functions including cell death, cell proliferation and differentiation and immune response via the MAPK pathway, which is accompanied by an increase in intracellular ROS levels [48]. It has been reported that increased ASK1 activity is involved in myocardial fibrosis and apoptosis in Dox-induced cardiomyopathy [49], ischemic heart disease [50] and dilated cardiomyopathy [51]. Interestingly, Trx forms a complex with ASK1 in the cell, resulting in the suppression of ASK1 activity and the negative regulation of JNK and p38MAPK signals, which are downstream of ASK1 [52]. Therefore, Trx is positioned as an endogenous suppressor of ASK1 activity. Considering the fact that the amount of Trx in myocardial tissue was maintained by the administration of HSA-Trx (Figure 2B), it may be possible that the suppression of ASK1 activity could also be involved in the action of HSA-Trx. Further investigations will be needed regarding this.

An increased expression of myocardial atrophy factors atrogin-1 and myostatin was observed by Dox administration, and these increased expressions were suppressed by the administration of HSA-Trx. It has been reported that the inhibition of the atrogin-1 signal improves myocardial atrophy [21] and that myocardial-specific overexpressed myostatin induces myocardial atrophy [41,42]. Therefore, it appears that the suppression of atrogin-1 and myostatin expression caused by the administration of HSA-Trx was largely involved in the improvement in myocardial atrophy. It is generally known that atrogin-1 and myostatin expression is increased by oxidative stress and inflammatory cytokines such as TNF-a. This suggests that the regulation of myocardial atrophy factor expression by HSA-Trx may be due to the suppression of oxidative stress and inflammatory reactions in myocardial tissue. This study did not investigate the myocardial energetic network as a target of Dox toxic action in the heart. In a future investigation, a direct protective effect of HSA-Trx on Dox-induced mitochondrial dysfunction would be needed [36].

## 5. Conclusions

**Figure 5:**
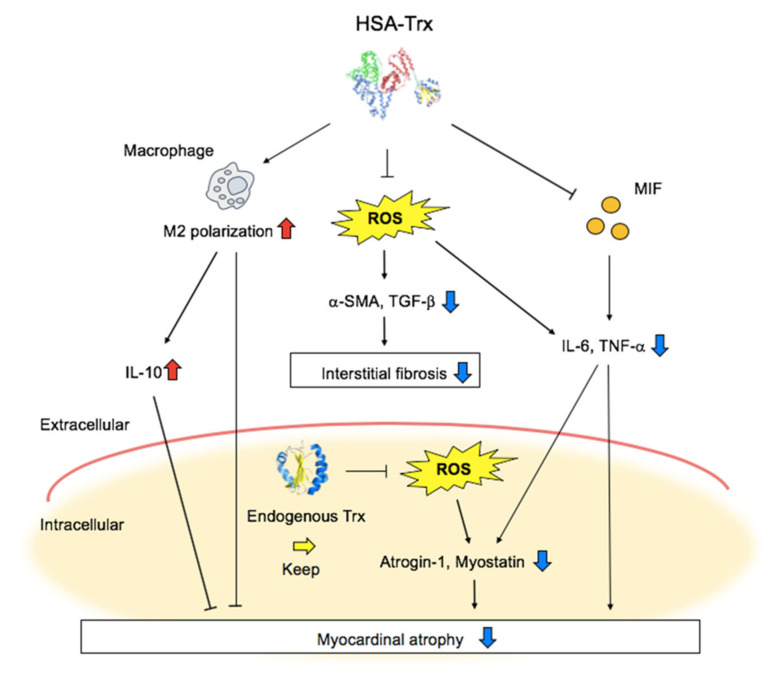
Schematic diagram showing the effect of HSA-Trx on Dox-induced myocardial atrophy.

In this study, we provide data to show that HSA-Trx exerted anti-oxidative and anti-inflammatory effects, and showed an improving effect on the progression of heart failure such as Dox-induced cardiomyopathy and fibrosis (Figure 5). We therefore conclude that HSA-Trx could be a potential therapeutic agent targeting myocardial remodeling for the treatment of heart failure. In the future study, we need to obtain evidence on the protective effect of HSA-Trx on other animal models of heart failure such as cardiac ischemia–reperfusion injury and pressure overload-induced heart failure mice to strengthen the applicability of HSA-Trx.

## Data Availability

Not applicable.

## Supplementary Materials

The following are available online at https://www.mdpi.com/article/10.3390/pharmaceutics14030562/s1, Figure S1: Dox-induced cardiomyopathy model mice by the intraperitoneal injection of Dox; Figure S2: The effect of HSA-Trx on body weight decrease in Dox-induced cardiomyopathy model mice; Figure S3: Complete Western blot images before cropping into result Figure 2; Table S1: The primers used for mRNA detection.
