# Supplementary material for: Long-Acting Thioredoxin Ameliorates Doxorubicin-Induced Cardiomyopathy via Its Anti-Oxidative and Anti-Inflammatory Action
Source: Pharmaceutics. 2022 Mar 3;14(3):562. doi: 10.3390/pharmaceutics14030562 (PMC8953310; doi:10.3390/pharmaceutics14030562)
Supplement: Supplementary file 1 [file pharmaceutics-14-00562-s001.zip › pharmaceutics-1576723-supplementary.pdf]

pharmaceutics

MDPI

Supplementary Materials: Long-Acting Thioredoxin Ameliorates Doxorubicin-Induced Cardiomyopathy via Its Anti-Oxidative and Anti-Inflammatory Action

Ryota Murata, Hiroshi Watanabe, Hiroto Nosaki, Kento Nishida, Hitoshi Maeda, Motohiro Nishida and Toru Maruyama

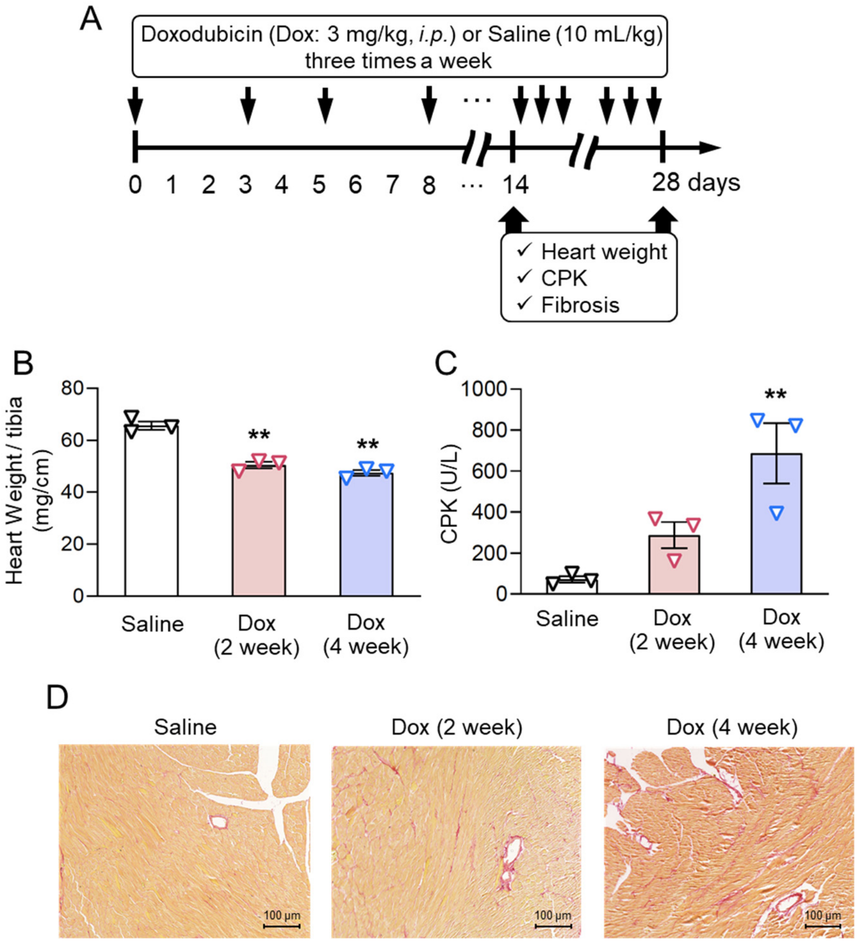

Figure S1. Dox-induced cardiomyopathy model mice by the intraperitoneal injection of Dox. (**A**) Experimental protocol for the Dox-induced cardiomyopathy model mice by the intraperitoneal injection of 3 mg/kg Dox three times per week for 4 weeks. (**B**) Changes in heart weight after the injection of Dox. (**C**) Change in the level of creatine phosphokinase (CPK). (**D**) Sirius red-stained heart sections at 2 and 4 weeks after the first injection of Dox. Original magnification: ×200. Scale bars represent 100 μm. Data are expressed as the mean±SE, $^{**}p<0.01$.

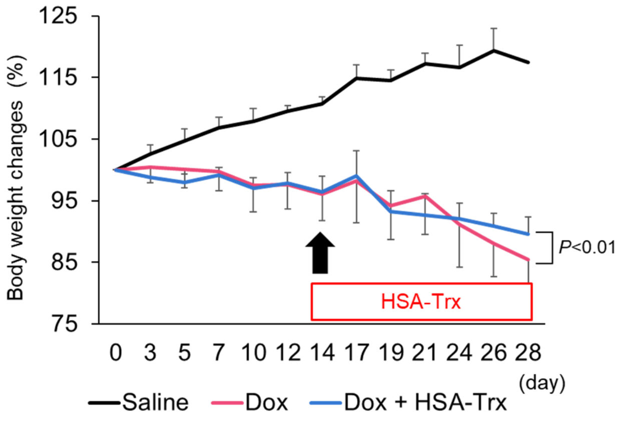

Figure S2. The effect of HSA-Trx on body weight decrease in Dox-induced cardiomyopathy model mice. Dox-induced cardiomyopathy was induced by an intraperitoneal injection of 3 mg/kg Dox three times a week for 4 weeks. HSA-Trx (400 nmol/kg) was administered *i.v.* three times per week at 2 weeks after the first injection of Dox. Data are expressed as the mean±SE.

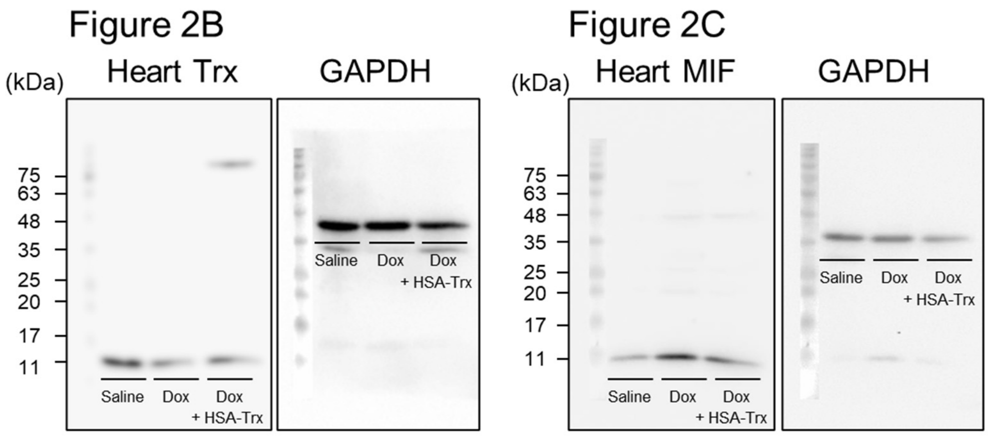

Figure S3. Complete Western blot images before cropping into result Figure 2.

Table S1. The primers used for mRNA detection.

Target gene	Forward (5′→3′)	Reverse (5′→3′)
GAPDH	AACTTTGGCATTGTGGAAGG	ACACATTGGGGGTAGGAACA
α-SMA	AGCCATCTTTCATTGGGATGG	CCCCTGACAGGACGTTGTTA
TGF-β	GGATACCAACTATTGCTTCAGCTCC	AGGCTCCAAATATAGGGGCAGGGTC
TNF-α	CATGAGCACAGAAAGCATGATCCG	AAGCAGGAATGAGAAGAGGCTGAG
IL-6	TCTCTGCAAGAGACTTCCATCC	AGACAGGTCTGTTGGGAGTG
IL-10	GGACAACATACTGCTAACCGACTC	AAAATCACTCTTCACCTGCTCCAC
iNOS	GTGGTGACAAGCACATTTGG	AAGGCCAAACACAGCATACC
CD206	GCCAGAGACATAACAGCA	CAGGTTTCCTTTCAGTCCT
Atrogin-1	CAGAGAGGCAGATTCGCAAG	GGTGACCCCATACTGCTCTC
Myostatin	CTGTAACCTTCCCAGGACCA	TCTTTTGGGTGCGATAATCC
PGC-1α	TCTCAGTAAGGGGCTGGTTG	AGCAGCACACTCTATGTCACTC
